# An Image Dataset Quality Evaluation System for Industrial Object Detection Tasks

**DOI:** 10.3390/s26144465

**Published:** 2026-07-14

**Authors:** Shengguo Zhu, Yunxi Sun, Enhui Lu, Xinglong Zhu, Jian Liu

**Affiliations:** 1 School of Mechanical Engineering, Yangzhou University, Yangzhou 225127, China; shengguozhu01@163.com (S.Z.); 13813451181@163.com (Y.S.); xlzhu@yzu.edu.cn (X.Z.); 2 State Key Laboratory of Advanced Design and Manufacture for Vehicle Body, Hunan University, Changsha 410082, China; liujian@hnu.edu.cn

**Keywords:** image dataset quality evaluation system, industrial object detection, dataset acquisition guidelines, Taguchi, deep learning

## Abstract

The performance of visual detection models in industrial applications is strongly influenced by training dataset quality. Although imaging scheme design and algorithm optimization are often emphasized, systematic dataset quality evaluation remains insufficient. To address this gap, this study proposes a dataset quality evaluation framework for industrial object detection. It includes four dimensions and thirteen quantifiable indices: three for acquisition environment, two for image quality, three for dataset scale, and five for annotation quality. Normalization based on theoretical maximum scores is used to reduce biases caused by different score ranges, and dimension weights are assigned using Taguchi orthogonal experiments. Validation is performed on five public and three self-constructed datasets using YOLOv12n and RT-DETR-R18. A positive correlation trend is observed between the proposed scores and detection accuracy, with PLCC/SRCC/Kendall’s tau values of 0.685/0.850/0.764 and 0.656/0.826/0.691, respectively. After second-level weight optimization, the correlations increase to 0.775/0.922/0.837 and 0.748/0.898/0.764. Corresponding *p*-values and 95% confidence intervals are reported to quantify statistical uncertainty. Sensitivity analysis and ablation comparisons further verify the robustness and necessity of the proposed multidimensional framework. The proposed framework provides a quantifiable method and practical acquisition guidelines for improving industrial image dataset quality.

## 1. Introduction

With the ongoing advancement of industrial technology and growth in application demands, significant improvements have been observed in the structural complexity and automation of modern industrial systems. In this context, data-driven methods, especially computer vision techniques, have emerged as key approaches for industrial detection and quality control. However, the performance of these methods depends heavily on the quality of training datasets, which are considered a critical factor influencing model reliability and safety.

In practical industrial scenarios, issues such as variations in the acquisition environment, unstable image quality, uneven data distribution, and annotation errors can compromise model detection performance and prediction accuracy. These problems may also jeopardize the stable operation- and safety-related decision-making of industrial systems, potentially resulting in false detections and missed detections. Therefore, establishing a scientific, objective, and quantifiable evaluation index system for image datasets is crucial for enhancing model training performance, improving system robustness, and fostering the development of industrial intelligence.

Currently, traditional industrial detection relies on human experience, complicating real-time and large-scale processing and resulting in a lack of unified evaluation standards. Significant advancements in machine vision-based object detection have addressed numerous issues associated with manual methods, focusing on imaging design [1], dataset construction [2], algorithm optimization [3], performance metrics [4], and task-specific challenges [5]. However, most studies remain model-centered [6], concentrating on optimizing detection network structures, enhancing feature fusion, and ensuring lightweight deployment. For example, Lu et al. [7] developed WSS-YOLO, incorporating WIoU loss, dynamic snake convolution, and a lightweight neck structure, achieving mAP values of 82.3% and 72.0% on the NEU-DET and GC10-DET datasets, and confirming its effectiveness in industrial defect detection. Liang et al. [8] proposed a detection framework using Siamese self-supervised pretraining and multi-scale feature enhancement, attaining an mAP of 77.84%, showcasing its accuracy and generalization in complex scenarios. Similarly, Zhang et al. [9] designed DsP-YOLO for detecting multi-scale small defects in complex backgrounds, improving detection accuracy by 3.6%, 2.1%, and 3.9% on the NEU-DET, PCB-DET, and GC10-DET datasets, respectively, compared to the original YOLOv8 model.

In contrast, systematic and quantifiable methods for dataset evaluation remain underexplored. Majeed et al. [10] proposed an alternative approach called data-centric artificial intelligence, focusing on dataset construction. Recently, there has been growing attention on object detection dataset creation and the impact of dataset quality on algorithm performance. Mohanty et al. [11] demonstrated that models trained on data from controlled environments achieved an accuracy of 99.35% under experimental conditions, but this dropped to approximately 31% in real-world settings. This highlights how variations in acquisition environments can introduce dataset bias and affect model generalization. Zhou et al. [12] constructed industrial image datasets with 3000 distorted images, named ISID and IPID, and proposed the SIFS method for assessing image fidelity based on the human visual system, achieving a maximum PLCC of 0.8739, which significantly outperformed traditional methods. Kanwal et al. [13] noted that increasing the sample size from about five to over 15 groups shifted algorithm performance from statistically insignificant (Z < 1.96) to significant (Z > 1.96), indicating that evaluation results are highly dependent on dataset scale. Oksuz et al. [14] showed that on the PASCAL VOC dataset, the mAP of object detection models rose from 0.34 to roughly 0.80, though the significant imbalance of “a small number of positive samples against millions of negative samples” still affected performance. Bär et al. [15] introduced noisy labels into the Pascal VOC 2012 dataset and optimized them for Cascade R-CNN training, finding that mAP accuracy correlated positively with label quality. Murrugarra-Llerena et al. [16] found that increased annotation noise in mainstream datasets like COCO significantly reduced AP performance, particularly for small objects, with the gap between conventional AP and the noise-aware lower-bound AP potentially exceeding 20%.

In summary, existing studies are mostly limited to conducting qualitative evaluations of datasets from a single dimension, leading to issues such as partiality, lack of intuitiveness, and insufficient systematic analysis. The quantitative assessment of dataset quality remains insufficient. In response to these limitations, a multidimensional quality evaluation system for industrial object detection image datasets has been constructed in this study. The proposed system is based on first-level indices established from four dimensions: acquisition environment, image quality, dataset scale, and image annotation. Additionally, thirteen computable second-level indices have been designed. A normalization method based on theoretical maximum scores is then employed to mitigate the influence of discrepancies in full scores across dimensions, while Taguchi orthogonal experiments are utilized to determine the weights of each dimension. Five public industrial datasets and three self-constructed datasets are selected for experimental validation. The correlation between comprehensive evaluation scores and the detection performance of YOLOv12n and RT-DETR-R18 is analyzed to verify the effectiveness of the proposed evaluation system. Finally, guidelines for dataset acquisition are provided to establish evaluation standards for constructing quality-controllable industrial image datasets.

The proposed framework is designed as a dataset quality evaluation method rather than a regression model for directly predicting detector mAP. Since all indices are calculated from intrinsic dataset attributes, the framework can be applied to other industrial object detection datasets for quality assessment and comparison before model training. The downstream detection experiments in this study are used to examine whether the proposed quality score is associated with model performance, thereby providing preliminary validation of the framework.

Several indices adopted in this work, including PIQE, MOS, IoU-based localization accuracy, entropy-based background description, and sample balance, have been previously studied in image quality assessment, object detection evaluation, or dataset analysis. However, these indices are usually used separately for specific evaluation purposes. For industrial object detection datasets, dataset quality is jointly affected by acquisition environment, image quality, dataset scale, and image annotation. Therefore, the main novelty of this study lies in integrating existing and task-adapted indices into a unified, multidimensional, and quantifiable evaluation framework. Through directional normalization, comprehensive scoring, Taguchi-based weight analysis, and downstream model validation, the proposed framework provides a systematic method for evaluating and comparing industrial object detection datasets.

The main highlights of this study are as follows:

(1) Eight representative industrial object detection datasets are collected, including five public and three self-constructed datasets.
(2) A multidimensional and quantifiable dataset quality evaluation system is designed for industrial object detection datasets.
(3) YOLOv12n and RT-DETR-R18 are employed as downstream detection models to analyze the correlation between the comprehensive dataset scores and detection accuracy.
(4) Practical dataset acquisition guidelines are provided based on the proposed evaluation framework and Taguchi analysis.

The structure of this paper is organized as follows: Section 2 introduces the dataset construction process and describes the sources, composition, and basic information of the public and self-constructed datasets. Section 3 constructs the dataset quality evaluation system by establishing thirteen second-level indices based on four first-level indices: dataset acquisition environment, image quality, dataset scale, and image annotation quality. Section 4 presents data validation and guidelines, including calculation of normalized dataset scores, correlation validation and dataset acquisition guidelines. Section 5 draws the main conclusions.

## 2. Dataset Construction

**Figure 1:**
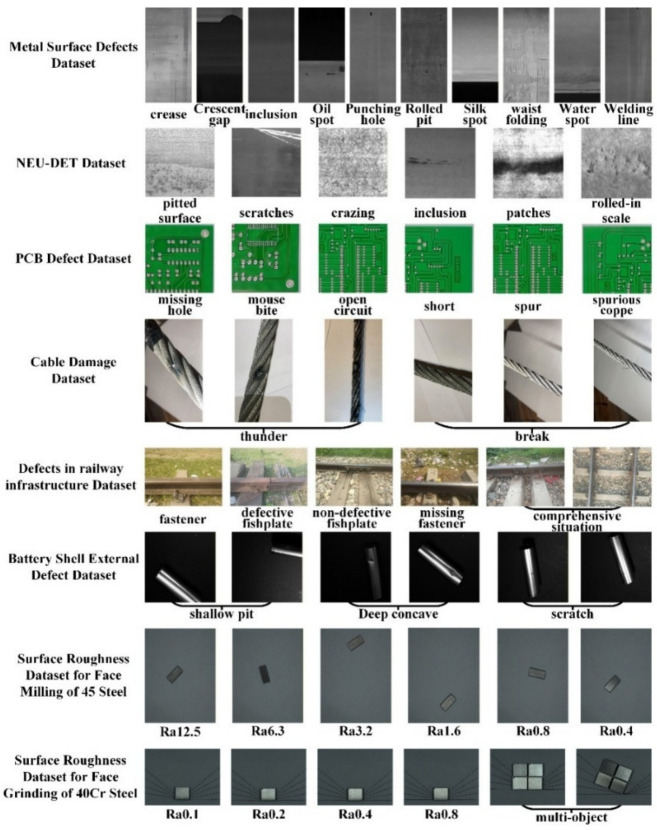
Example images of the related industrial datasets used in this study.

In this study, five public industrial datasets and three self-constructed datasets are collected and organized to support defect detection research across various industrial scenarios [17]. The public datasets encompass a range of categories, including cable damage, missing railway components, metal surface defects, and PCB defects, with image counts varying from 1318 to 8001; some of them provide predefined training, validation, and test splits. These public datasets are characterized by diversity and reusability, though some exhibit lower image quality and annotation accuracy. In contrast, the self-constructed datasets are smaller in scale but feature high annotation accuracy and clear images, making them suitable for training models in specific industrial contexts. Example images from the datasets are presented in Figure 1, while Table 1 provides detailed information about the datasets, including the number of categories, sample sizes, and data sources.

The datasets used in this study are heterogeneous in terms of object type, number of categories, image resolution, target scale, and detection difficulty. This heterogeneity reflects the diversity of industrial object detection scenarios, but it also means that downstream detection accuracy may be influenced by both dataset quality and intrinsic task difficulty. Therefore, the selected datasets are used to examine the relationship between dataset quality scores and detection performance under representative heterogeneous industrial conditions, rather than to completely isolate the effect of dataset quality from all task-difficulty factors.

Although the eight datasets contain 41 categories and 20,811 images in total and cover multiple industrial scenarios, the validation unit in this study is the dataset rather than individual images. Therefore, the effective sample size for correlation analysis is eight. The selected datasets are used to provide a preliminary validation of the proposed framework across different industrial scenarios, while further validation on more datasets is still needed to examine its broader generalizability.

## 3. Dataset Quality Evaluation System and Evaluation Index Construction

The indices used in this framework include both established metrics from previous studies and task-adapted metrics designed according to the characteristics of industrial object detection datasets. The purpose of this section is not to redefine each individual metric as a new index, but to organize them into a unified evaluation system with consistent score direction, normalized value ranges, and clear task relevance. To ensure the constructed evaluation system is applicable to industrial object detection datasets, the following principles are followed in the index design process:

(1) **Task Relevance**: The selected indices are closely related to the training and inference processes of object detection models, reflecting key factors that affect detection accuracy, convergence stability, and generalization capability.

(2) **Computability**: The indices are directly calculable based on image files, annotation files, acquisition parameters, or manual scores, avoiding reliance on subjective experience, which is difficult to obtain.

(3) **Interpretability**: Each index corresponds to a clearly defined dataset quality issue, such as insufficient samples, class imbalance, illumination fluctuations, background interference, image distortion, or annotation errors.

(4) **Scalability**: The index system is applicable to various industrial detection scenarios, with reference thresholds and weight parameters adjustable according to specific tasks.

(5) **Directional Consistency**: After normalization, all indices are uniformly defined so that a larger value indicates higher dataset quality, facilitating subsequent comprehensive scoring.

**Figure 2:**
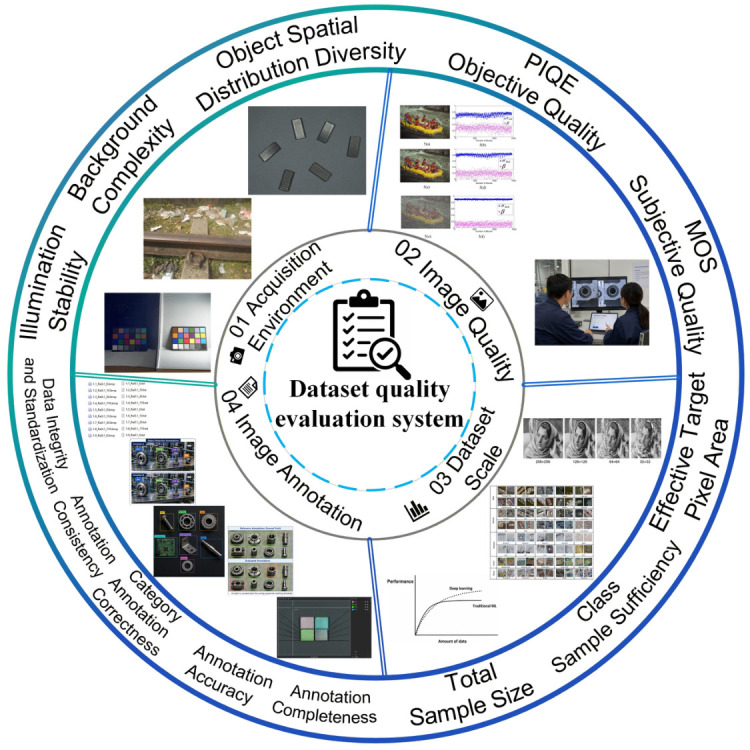
Index system and relationships among indices.

The overall framework and details are presented in Figure 2 and Table 2, respectively. The scores of all indices increase with better dataset quality; therefore, all indices are positively oriented.

For indices involving multiple sub-features, the corresponding fusion coefficients are used as normalized intra-index weights, rather than weights among different evaluation indices. These coefficients are introduced to combine sub-features with different physical meanings into a unified score within the same index. Unless otherwise specified, they are selected according to task relevance, interpretability, and their influence on dataset usability, and they can be recalibrated for specific industrial scenarios.

### 3.1. Acquisition Environment

#### 3.1.1. Illumination Stability Score

The illumination condition of a dataset is quantified by calculating the average brightness of each image. For RGB images, the brightness value is calculated as a weighted sum of the color channels [19]. The average brightness of the dataset is obtained by averaging the brightness values of all images. Additionally, the standard deviation of image brightness is calculated to assess illumination stability.

The influence of overall brightness differences across datasets on illumination stability evaluation is eliminated by utilizing the coefficient of variation, defined as the ratio of brightness standard deviation to mean brightness. This normalized measure reflects the relative fluctuation of image brightness. When brightness fluctuation is minimal, a relatively small standard deviation compared to the mean brightness results in a higher score for illumination stability. The calculation method is shown in Equations (1)–(3):

(1) $$\mu_{L}=\frac{1}{N}\sum_{i=1}^{N}L_{i}$$

(2) $$\sigma_{L}=\sqrt{\frac{1}{N}\sum_{i=1}^{N}{\left(L_{i}-\mu_{L}\right)}^{2}}$$

(3) $$S_{illum}=1-\frac{\sigma_{L}}{\mu_{L}}$$

Here, $L_{i}$: the average brightness of the (i)−th image, $\mu_{L}$: the mean brightness of all images, $\sigma_{L}$: the brightness standard deviation, and *N*: the number of images. Specifically, $S_{illum}\approx 1$ indicates highly stable illumination, $S_{illum}\approx 0.7-0.9$ indicates relatively stable illumination suitable for industrial applications, and $S_{illum}<0.5$ indicates obvious illumination fluctuation.

#### 3.1.2. Background Complexity

Background complexity is comprehensively characterized by modeling it from three aspects: grayscale statistical characteristics, spatial structural information, and texture details. Information entropy, gradient energy, and the proportion of high-frequency components are utilized as the corresponding measurement indices. To mitigate the interference of texture and edge information in target regions on background complexity evaluation, foreground masks are constructed based on annotation information, and target regions are removed from the images. Feature calculation is then performed solely on the background regions, enhancing the accuracy and specificity of the evaluation results. This strategy more accurately reflects the texture complexity and interference level of the background regions in the dataset. Since different features have different dimensions and numerical ranges, a unified background complexity evaluation model is constructed through weighted fusion, as defined in Equations (4)–(7):

(4) $$H=-\sum_{i=0}^{255}p_{i}\log_{2}(p_{i})$$

(5) $$G=\frac{1}{N}\sum |\nabla I(x,y)|$$

(6) $$F=\frac{\sum |I_{high}|}{\sum I}$$

(7) $$S_{bg}=1-\left(w_{1}\frac{H}{H_{\max}}+w_{2}\frac{G}{G_{\max}}+w_{3}\frac{F}{F_{\max}}\right)$$

Here, *N*: the total number of pixels in the image; normalized reference upper bounds are defined for each index corresponding to $H_{\max}$, $G_{\max}$ and $F_{\max}$. H represents image grayscale information entropy, G represents average gradient magnitude, and F represents high-frequency response intensity; for 8-bit grayscale images, the maximum entropy, $H_{\max}$, is set to 8, while $G_{\max}=\max\left(G_{\mathrm{mean}}\right)$, and $F_{\max}=\max\left(F_{\mathrm{mean}}\right)$ are set to ensure normalization stability.

The three fusion coefficients are constrained to be non-negative and to satisfy $w_{1}+w_{2}+w_{3}=1$. Entropy (*H*), average gradient magnitude (*G*), and high-frequency response intensity (*F*) represent different types of background interference, namely grayscale distribution complexity, structural edge interference, and texture-detail interference, respectively. In industrial object detection, background edge structures usually have a more direct influence on target boundary feature extraction; therefore, the gradient term is assigned a relatively higher weight.

In this study, particle swarm optimization is used to search for an appropriate combination of $w_{1}$, $w_{2}$, and $w_{3}$ under the above normalization constraint. When the weights are set to $w_{1}$ = 0.3, $w_{2}$ = 0.4, and $w_{3}$ = 0.3, the background complexity score shows a relatively strong correlation with the detection performance. This setting gives slightly higher priority to structural edge interference while still preserving the contributions of grayscale entropy and high-frequency texture details. Therefore, this weight combination is adopted in this study. It should be noted that these weights are recommended values for the datasets investigated in this work and can be recalibrated for other industrial scenarios.

Finally, the background complexity score $S_{bg}$ is defined such that a larger value indicates a simpler background and less interference.

#### 3.1.3. Object Spatial Distribution Diversity

To comprehensively characterize the spatial distribution diversity of targets in images, target spatial distribution is modeled from three aspects: dispersion, coverage, and uniformity. First, the target center coordinates are normalized to the interval [0, 1] to eliminate the influence of different image sizes and aspect ratios. Second, the standard deviations of the horizontal and vertical coordinates are used to characterize the dispersion degree of target distribution. Furthermore, the normalized coordinate space is divided into Z×Z grids, and the proportion of grids occupied by targets is calculated to measure spatial coverage, thereby reflecting the distribution breadth of targets within the field of view. Meanwhile, spatial entropy is introduced to characterize the distribution uniformity of targets in different regions. When targets are more uniformly distributed across grids, the corresponding entropy value is higher. Based on the above three indices, a comprehensive object spatial distribution diversity score is constructed, as shown in Equation (8):

(8) $$S_{view}=\psi_{1}\cdot \frac{\sqrt{\sigma_{x}^{2}+\sigma_{y}^{2}}}{\sqrt{2}}+\psi_{2}\cdot C+\psi_{3}\cdot \frac{h_{s}}{h_{\max}}$$

Here, C denotes grid coverage, $h_{s}$ denotes spatial distribution entropy, and $h_{\max}$ is maximum entropy. The weights of the three components are set according to their roles in object detection. The coverage term reflects whether objects appear in a sufficiently wide range of image regions, which directly affects the model’s ability to learn objects under different spatial positions and viewpoints. Therefore, it is assigned the highest weight. The normalized standard deviation term and spatial entropy term describe the dispersion and uniformity of object locations, respectively. Since these two components provide complementary information, they are assigned the same auxiliary weight. Thus,$\psi_{1}$ = 0.3, $\psi_{2}$ = 0.4, and $\psi_{3}$ = 0.3 are used in this study [20,21]. These coefficients are not intended to be universal constants and may be adjusted according to the spatial distribution characteristics of specific datasets. This index can comprehensively reflect the object spatial distribution characteristics of targets from multiple dimensions and has stronger descriptive capability than a single statistical measure.

### 3.2. Image Quality

To quantitatively evaluate the visual quality of different image datasets, this study constructs an image quality analysis method from both objective and subjective perspectives. The objective evaluation adopts the perception-based no-reference image quality assessment index PIQE, namely the Perception-based Image Quality Evaluator. The subjective evaluation adopts the mean opinion score, namely MOS.

#### 3.2.1. MOS Subjective Score

To further reflect human visual perception in subjective judgments of image quality, dataset images are manually evaluated using the mean opinion score (MOS). MOS [22] is a commonly used subjective evaluation index in the field of image quality assessment, as it comprehensively reflects observers’ subjective perceptions of image clarity, noise level, structural integrity, target distinguishability, and visual comfort.

Six observers are invited to participate in the MOS scoring experiment. All observers have normal or corrected-to-normal vision and received unified scoring instructions before the evaluation. During scoring, the same number of image samples is randomly selected from each dataset, and the image order is randomized before being presented to the observers. The observers independently scored the images without knowing the image source, dataset name, category label, or objective quality scores, so as to reduce the influence of prior information on subjective judgment. All images are evaluated using the same display device and brightness conditions. The scoring range is set from 1 to 5, where 1 indicates poor image quality and 5 indicates excellent image quality.

It should be noted that the number of observers used for MOS scoring in this study is limited. Therefore, the MOS result is regarded as an auxiliary subjective quality estimate rather than a large-scale psychophysical evaluation. In this study, MOS is used together with the objective PIQE index to jointly characterize image quality, thereby avoiding reliance on subjective scoring alone. The MOS is expressed in Equation (9):

(9) $$MOS=\frac{1}{N}\sum_{i=1}^{N}S_{i}$$

where $S_{i}$ denotes the score given by the (i)−th observer, and *N* denotes the number of observers.

Since the value range of MOS is [1, 5], a min–max normalization method is adopted to map it to the interval [0, 1] so as to unify the scale of different indices and eliminate dimensional effects, as shown in Equation (10):

(10) $$S_{MOS}=\frac{MOS-1}{4}$$

After normalization, $S_{MOS}$ closer to 1 indicates higher subjective image quality, whereas a value closer to 0 indicates lower subjective image quality.

#### 3.2.2. PIQE Objective Quality

PIQE [23] is used as a typical no-reference image quality assessment method, by which the visual quality of an input image is automatically evaluated without an original undistorted reference image. PIQE is selected as the objective index in this study, mainly because undistorted reference images are generally not provided in industrial detection datasets.

Given an input image, it is firstly divided into *N* local image blocks by PIQE, and the distortion degree of each block is computed. The overall raw PIQE score can be expressed as Equation (11):

(11) $$PIQE=\frac{1}{N}\sum_{i=1}^{N}D_{i}$$

Here, $D_{i}$: distortion degree of the (i)−th image block, and N: the total number of image blocks. The raw PIQE score generally falls within the range of 0–100, where a smaller value indicates less image distortion and better visual quality. To make its numerical direction consistent with the subsequent subjective MOS, this study converts the raw PIQE score into a quality score within the interval [0, 1], as shown in Equation (12):

(12) $$S_{PIQE}=1-\frac{PIQE}{100}$$

Here, $S_{PIQE}$ represents normalized objective image quality score ranges from 0 to 1. A value closer to 1 indicates higher image quality, whereas a value closer to 0 indicates more severe image distortion.

For analyzing the consistency between objective indices and subjective perception, the Pearson linear correlation coefficient (PLCC) [24,25] and the Spearman rank correlation coefficient (SRCC) [26] are employed for correlation analysis in this study. In this study, PLCC is used to measure the linear correlation between comprehensive scores and detector mAP, while SRCC is used to measure their ranking consistency. The closer the two values are to 1, the stronger the consistency between the comprehensive dataset evaluation results and model detection performance.

The PLCC and SRCC values are computed as 0.640 and 0.595, respectively, suggesting a moderate degree of consistency between the PIQE objective quality evaluations and the MOS subjective scores. A certain linear correlation is observed between PIQE scores and MOSs from the PLCC result, while the SRCC result shows that PIQE aligns reasonably well with the MOS subjective ranking when judging image quality across different datasets. Since only eight datasets are selected for correlation analysis in this study, the sample size is considered relatively small. Therefore, the correlation results are mainly used to support the feasibility of using PIQE as an objective image quality descriptor, rather than as evidence that PIQE can completely replace MOS. In summary, PIQE enables rapid, reference-free objective quantification of dataset image quality, and demonstrates a fair degree of consistency with MOS subjective scores. The moderate consistency between PIQE and MOS also indicates that MOS provides useful subjective information, but it should be interpreted as a complementary index due to the limited number of observers.

### 3.3. Dataset Scale

#### 3.3.1. Effective Target Pixel Area

For object detection tasks, the resolution of the entire image is not considered sufficient to fully reflect the distinguishability of targets. This is especially true in industrial defect detection, where defect targets are typically small. Even when the original image is provided with a high resolution, stable features may still be difficult for the model to extract if the target region occupies only a small number of pixels. Therefore, the pixel area of target bounding boxes is used in this study as the basis for effective resolution evaluation, with the specific calculation being shown in Equations (13)–(15):

(13) $$A_{i}=w_{i}\cdot h_{i}$$

(14) $$\bar{A}=\frac{1}{M}\sum_{i=1}^{M}A_{i}$$

(15) $$S_{res}=min\left(1,\frac{\bar{A}}{A_{ref}}\right)$$

where $w_{i}$ and $h_{i}$ represent pixel width and pixel height of the (i)−th target bounding box; M denotes the total number of targets in the dataset; $\bar{A}$ represents the median pixel area of targets in the dataset; and $A_{ref}$ denotes the reference target area threshold. When $\bar{A}\geq A_{ref}$, the dataset is considered to reach an ideal level in terms of target resolution, and the score is set to 1. Otherwise, the score changes linearly with the median target area.

In this study, the reference target area $A_{ref}$ is set to 1024, corresponding to a target region of 32×32 pixels [27]. This value is used as a reference scale for evaluating whether the target region contains sufficient effective visual information for feature extraction. In industrial object detection, especially in defect detection and small-object detection tasks, targets with too few pixels may lose important edge, texture, and shape information, even when the original image resolution is high. Therefore, a 32×32 pixel target region is adopted in this study as a relatively conservative reference area for comparing the effective target resolution of different datasets.

It should be noted that $A_{ref}$ = 1024 pixels is not a universal constant for all industrial detection tasks. The appropriate reference area depends on the target size, image resolution, input resizing strategy, detector architecture, and the minimum recognizable defect size in a specific application. Therefore, $A_{ref}$ can be recalibrated according to task requirements. For micro-defect detection, a task-specific threshold may be determined according to the minimum recognizable defect size, whereas for large-component detection, a larger reference area may be more appropriate. In this study, $A_{ref}$ = 1024 pixels is used as a unified reference value to ensure comparability among the selected datasets.

#### 3.3.2. Class Sample Sufficiency

This index measures class distribution balance rather than absolute sample count. Therefore, it is used together with the total sample size score to reflect both sample sufficiency and class balance. A higher score is obtained when per-class sample counts are closer to the average, while a lower score results from significantly under- or over-represented classes, as shown in Equation (16). In industrial detection tasks, the number of classes *K* is typically small, but some classes may be severely under-represented, leading to model bias.

(16) $$S_{cls}=1-\frac{1}{K}\sum_{i=1}^{K}\left|\frac{n_{i}-\bar{n}}{\bar{n}}\right|$$

where *K* denotes the total number of classes, $n_{i}$: the number of samples in the (i)−th class, $\bar{n}$: the average number of samples per class, and $\left|\frac{n_{i}-\bar{n}}{\bar{n}}\right|$: the inter-class difference ratio.

#### 3.3.3. Total Sample Size Score

Since dataset requirements vary significantly across tasks, the reference benchmark scale is not fixed [28,29,30] but is determined experimentally. This reflects a common pattern in visual recognition tasks: when the number of samples per class exceeds several hundred, further gains in model performance gradually saturate, as shown in Equations (17)–(19). Based on existing studies and industrial practice, selecting 500–1000 samples per class is generally considered to achieve a reasonable balance between data sufficiency and annotation cost.

(17) $$N_{ref}=\alpha \cdot C$$

(18) $$N_{eff}=\frac{{\left(\sum_{i=1}^{C}n_{i}\right)}^{2}}{\sum_{i=1}^{C}n_{i}^{2}}$$

(19) $$S_{vol}=min\left(1,\frac{\ln\left(N_{eff}+1\right)}{\ln\left(N_{ref}+1\right)}\right)$$

Here, $N_{ref}$: the reference benchmark scale, α: the recommended number of samples per class, *C*: the number of classes, and *N*: the total number of images in the dataset.

### 3.4. Image Annotation

**Figure 3:**
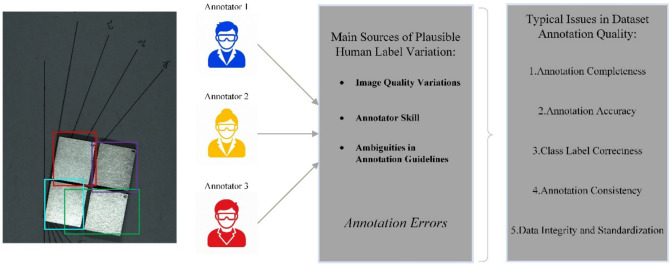
Schematic diagram of annotation quality issues and evaluation indices for object detection datasets.

In object detection tasks, dataset annotation quality [31,32] is mainly reflected in target localization, instance coverage, class assignment, and consistency among multiple annotators. Due to inconsistent annotation standards, datasets usually suffer from problems such as deviations between bounding boxes and real targets, incorrect class labels, missed targets, redundant annotations, and erroneous merging or splitting of target instances. These problems reduce the model’s ability to learn target features and spatial information and affect detection accuracy and generalization performance. The specific problems are illustrated in Figure 3.

This study constructs an annotation quality evaluation index system for object detection datasets from five aspects: annotation completeness, annotation accuracy, category annotation correctness, annotation consistency, and data integrity and standardization. The first four aspects are mainly used to measure the reliability of annotation content itself, whereas the data integrity and standardization index is used to evaluate whether the dataset file structure, image–annotation matching relationship, annotation format, and naming rules meet the requirements of model training.

To balance evaluation representativeness with manual review cost, a stratified random sampling strategy is adopted to construct the annotation quality evaluation subset. For datasets containing fewer than 3000 images, 20% of the images are sampled; for larger datasets, no fewer than 500 images are sampled. During sampling, the class proportion is kept largely consistent with that of the original dataset. The reference annotations are obtained by the authors through manual review according to unified annotation specifications.

#### 3.4.1. Annotation Completeness

To further improve the comprehensiveness of annotation completeness evaluation, traditional matching-rate-based indices mainly consider missed annotations, while the influence of duplicate annotations on data quality is ignored. In actual datasets, excessive redundant annotations may also interfere with model training and degrade detection performance. Therefore, a duplicate-annotation penalty term is introduced based on the original completeness index, and the annotation completeness score is defined accordingly, as shown in Equation (20):

(20) $$S_{comp}=\frac{N_{m}}{N_{r}+\lambda N_{fp}}$$

where $N_{m}$: matched annotations; $N_{r}$: the total number of reference annotations; $N_{fp}$: the number of redundant annotations that are not matched with any reference target; and λ: the parameter used to control the penalty strength of redundant annotations on the completeness score. This study sets the λ to 0.5 to achieve a balance between missed-annotation penalty and duplicate-annotation penalty.

#### 3.4.2. Annotation Accuracy

An evaluation method based solely on IoU reflects only the localization accuracy of annotation boxes, without characterizing annotation completeness. In actual datasets, missed annotations or insufficient matching may also significantly affect model performance. Therefore, annotation accuracy is decomposed in this study into two aspects: localization accuracy and matching completeness. First, the average IoU is used to measure the localization accuracy of matched targets. Second, the matching rate is introduced to reflect the coverage of reference targets. The annotation accuracy score is then defined, as shown in Equations (21)–(23):

(21) $$S_{loc}=\frac{1}{N_{m}}\sum IoU_{i}$$

(22) $$S_{rec}=\frac{N_{m}}{N_{r}}$$

(23) $$S_{conn}=S_{loc}\cdot S_{rec}=\frac{1}{N_{m}}\sum IoU_{i}\cdot \frac{N_{m}}{N_{r}}$$

Here, $N_{m}$: matched annotations, and $N_{r}$: the total number of reference annotations. The closer the IoU is to 1, the more consistent the original bounding box is with the reference bounding box. When IoU is greater than 0.5, the two bounding boxes are considered successfully matched.

#### 3.4.3. Category Annotation Correctness

Category annotation correctness is used to evaluate the accuracy of category labels among matched targets. For all successfully matched targets, the consistency between their assigned categories and the reference annotations is counted. To reduce the influence of class imbalance, class-wise accuracy is further introduced, and a weighted average is performed, as shown in Equations (24) and (25).

(24) $$w_{k}=\frac{1/f_{k}}{\sum_{j=1}^{K}\left(1/f_{j}\right)}$$

(25) $$S_{cat}=\sum_{k=1}^{K}w_{k}\frac{N_{c}^{\left(k\right)}}{N_{m}^{\left(k\right)}}$$

where $N_{m}^{\left(k\right)}$: the number of matched targets in the (k)−th class, and $N_{c}^{\left(k\right)}$: the number of correctly labeled targets, where the weight $w_{k}$ is inversely proportional to class frequency. $f_{k}$. This design enhances attention to classes with fewer samples, thereby improving evaluation fairness.

#### 3.4.4. Annotation Consistency

To comprehensively evaluate annotation consistency, it is modeled in this study from two aspects: spatial-location consistency and category consistency. The traditional Cohen’s Kappa coefficient is mainly used to measure classification consistency, but it cannot reflect deviations in bounding box locations in object detection tasks. Spatial consistency is therefore defined as the average IoU between matched targets, while category consistency is measured using Cohen’s Kappa coefficient, as shown in Equations (26) and (27):

(26) $$\kappa =\frac{P_{o}-P_{e}}{1-P_{e}}$$

(27) $$S_{con}=\gamma S_{loc}+(1-\gamma )\kappa$$

Here, $P_{o}$: the observed agreement probability, $P_{e}$: the chance agreement probability, and $S_{loc}$ refers to the average IoU for matched targets. It is defined in accordance with Equation (22) and used to measure the consistency of object positions across different annotation results. γ: the weight coefficient. The parameter γ controls the relative contribution of spatial-location consistency and category consistency. In object detection tasks, both bounding-box localization consistency and class-label consistency are essential for reliable annotation quality. A localization-consistent but category-inconsistent annotation, or a category-consistent but poorly localized annotation, may negatively affect model training. Since there is no strong prior evidence that either component is consistently more important across the industrial datasets considered in this study, γ = 0.5 is adopted to give equal importance to spatial consistency and category consistency. This setting avoids introducing an artificial preference toward either localization or classification consistency.

#### 3.4.5. Data Integrity and Standardization

The data integrity and standardization index is used to evaluate dataset quality in terms of file structure, annotation matching, and format specifications. This study conducts quantitative modeling from four aspects: file integrity, image–annotation pairing completeness, format standardization, and naming consistency. File integrity is defined as the proportion of valid data, as shown in Equation (28). Image–annotation pairing completeness is defined as the one-to-one correspondence ratio between images and annotation files, as shown in Equation (29). Format standardization measures whether annotations conform to the standard format, as shown in Equation (30). Naming consistency is used to measure whether image files, annotation files, and class names follow unified naming rules, as shown in Equation (31).

(28) $$S_{file}=\frac{N_{valid}}{N_{total}}$$

(29) $$S_{pair}=\frac{N_{paired}}{N_{images}}$$

(30) $$S_{format}=\frac{N_{standard}}{N_{total}}$$

(31) $$S_{name}=\frac{N_{consistent}}{N_{total}}$$

Here, $N_{valid}$: number of valid files; $N_{paired}$: number of successfully paired image–annotation file pairs; $N_{standard}$: number of annotation files that conform to the standard format; $N_{consistent}$: number of files that satisfy the naming consistency rules; $N_{total}$: total number of files in the dataset; and $N_{images}$: total number of image files in the dataset.

The data integrity and standardization score is defined accordingly, as shown in Equation (32),

(32) $$S_{data}=\beta_{1}S_{file}+\beta_{2}S_{pair}+\beta_{3}S_{format}+\beta_{4}S_{name}$$

The weight parameters β are determined according to the influence of each sub-index on dataset usability and training stability. File integrity and image–annotation pairing completeness are regarded as basic prerequisites for model training, because missing files or unmatched image–annotation pairs may directly prevent the dataset from being correctly loaded or used. Therefore, relatively higher weights are assigned to these two terms. Format standardization and naming consistency mainly affect the readability, consistency, and automation of the data-processing workflow. Although their direct influence on model performance is relatively weaker, they are still important for dataset reproducibility and practical deployment. Therefore, the weights are set as β = (0.3, 0.3, 0.2, 0.2). This setting gives priority to data usability while also considering the requirements of dataset standardization [33]. These coefficients can be adjusted when a specific application has stricter requirements for format standardization or naming rules.

It should be noted that data integrity and standardization is mainly a prerequisite index for data usability and training-pipeline stability. This index reflects whether the dataset can be correctly loaded, managed, and reused, but it does not directly characterize visual separability, object scale, defect visibility, or intrinsic detection difficulty. Therefore, a higher value of this index indicates better data management quality and reproducibility, but it does not necessarily imply a monotonic improvement in mAP across heterogeneous datasets after necessary pre-processing.

## 4. Data Analysis and Discussion

### 4.1. Calculation of Normalized Dataset Scores

To ensure the comparability of experimental results across different datasets, the calculation of dataset evaluation index scores is carried out under a unified hardware and software environment, while the main hyperparameters of the detection model training are kept consistent. The specific experimental environment configuration and model training hyperparameters are shown in Table 3 and Table 4, respectively.

**Figure 4:**
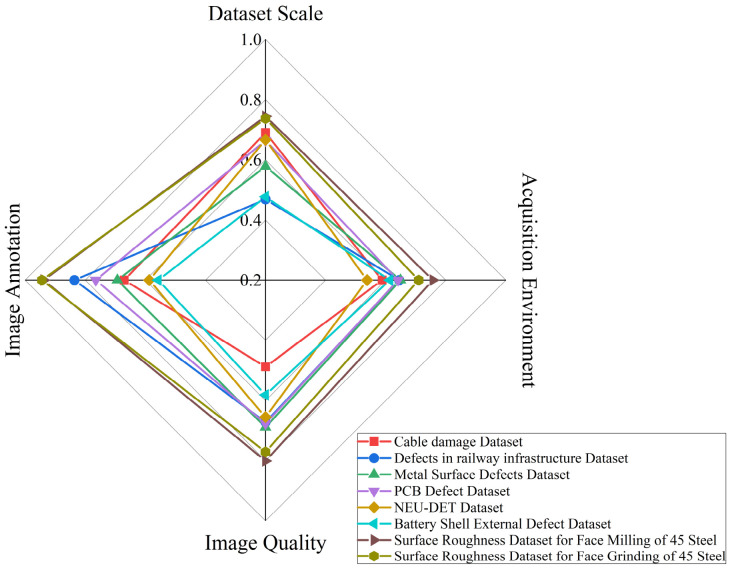
Radar charts of normalized scores for each dataset.

Since the dataset evaluation index system constructed in this study consists of four components—acquisition environment, image quality, dataset scale, and image annotation quality—the upper score limits of different indices are not identical. Therefore, before calculating the comprehensive scores of the first-level indices, each individual index is first normalized, as shown in Table 5. Specifically, the full scores for acquisition environment, image quality, dataset scale, and image annotation quality are 3, 2, 3, and 5, respectively. To preserve the absolute evaluation meaning of the indices and avoid excessive amplification of index differences caused by extreme values within the samples, a normalization method based on the theoretical maximum score is adopted in this study to map all indices uniformly into the [0, 1] interval. To make the first-level indices of each dataset more intuitive, radar charts of the normalized scores are plotted for each dataset, as shown in Figure 4.

### 4.2. Correlation Analysis

In this study, the mAP50 values of YOLOv12n [34] and RT-DETR-R18 [35] are selected as comparative evaluation indices. All datasets are cleaned and checked, and the training, validation, and test sets are divided at a ratio of 8:1:1. Since the four first-level indices have different degrees of influence on model performance, equal-weight summation may not fully reflect the importance of different quality factors. Therefore, the Taguchi orthogonal experimental method [36,37] is used to optimize the weights of each evaluation dimension. In the Taguchi method, each index weight is set to three levels, namely low, medium, and high, as shown in Table 6. An orthogonal experimental table $L_{9}\left(3^{4}\right)$ is constructed accordingly. For each weight combination, the comprehensive scores of the first-level evaluation indices for each dataset are first calculated according to the comprehensive evaluation model defined in the equation. Then, the detection accuracy of YOLOv12n on the corresponding dataset is used as the reference response, and the correlation between the dataset overall score and model accuracy is calculated. Finally, the weight combination with a higher correlation with YOLOv12n detection accuracy is selected as the recommended weight combination. The detection results of RT-DETR-R18 are used for cross-model validation. To comprehensively evaluate the consistency between the dataset overall score and model accuracy, PLCC, SRCC, and Kendall’s tau are used as evaluation metrics. To further quantify the uncertainty of the correlation results, 95% confidence intervals are calculated for PLCC, SRCC and Kendall’s tau. Considering that only eight datasets are used in this study, a non-parametric bootstrap strategy is adopted. Specifically, the dataset is used as the resampling unit, and eight paired samples of dataset quality score and model mAP50 are resampled with replacement in each bootstrap iteration. PLCC, SRCC and Kendall’s tau are then recalculated for each bootstrap sample. This process is repeated 10,000 times, and the 2.5th and 97.5th percentiles of the bootstrap distribution are used as the lower and upper bounds of the 95% confidence interval, respectively. The confidence intervals are used to evaluate the statistical uncertainty of the reported correlation coefficients.

**Figure 5:**
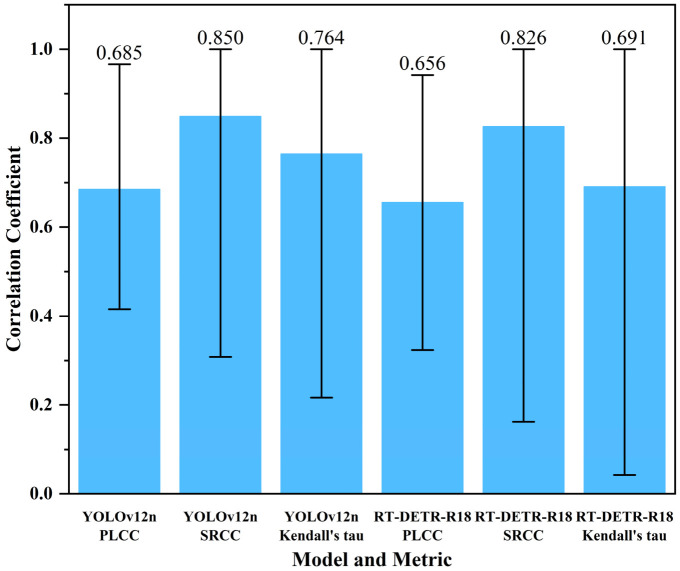
The 95% confidence intervals of PLCC, SRCC, and Kendall’s tau under the first-level weighting scheme.

Figure 5 shows the PLCC, SRCC, and Kendall’s tau values with 95% confidence intervals under the selected first-level weighting scheme. For YOLOv12n, the PLCC, SRCC, and Kendall’s tau are 0.685, 0.850, and 0.764, respectively, with corresponding *p*-values of 0.061, 0.007, and 0.009. For RT-DETR-R18, the PLCC, SRCC, and Kendall’s tau are 0.656, 0.826, and 0.691, respectively, with corresponding *p*-values of 0.078, 0.011, and 0.018. Although the PLCC *p*-values are slightly higher than 0.05, the positive PLCC values indicate a positive linear correlation trend. In contrast, SRCC and Kendall’s tau show positive rank correlations, indicating that the first-level comprehensive score has relatively strong ranking consistency with detection performance.

It should be noted that the correlation analysis is conducted at the dataset level with only eight datasets. In addition, multiple correlations and Taguchi-based weight searches are performed. Therefore, the reported *p*-values should be interpreted as nominal *p*-values and supplementary statistical references. The interpretation of the results is based on the correlation magnitudes, 95% confidence intervals, and the consistency of trends across YOLOv12n and RT-DETR-R18.

The definition of the dataset overall score of the first-level indices is given in Equation (33),

(33) $$S=w_{env}S_{env}+w_{quality}S_{quality}+w_{scale}S_{scale}+w_{ann}S_{ann}$$

where $w_{env}+w_{quality}+w_{scale}+w_{ann}=1$. To ensure the comparability among different weight combinations, the weight level coefficients of each group are normalized. Specifically, if the weight level coefficients of a certain experimental group are [*a,b,c,d*], the corresponding weight calculation method is shown in Equation (34):

(34) $$[w_{env},w_{quality},w_{scale},w_{ann}]=\frac{\left[a,b,c,d\right]}{a+b+c+d}$$

As shown in Table 7, the fourth weight combination achieves the highest overall response value among the $L_{9}\left(3^{4}\right)$ orthogonal experiments. The corresponding uncertainty visualization is presented in Figure 5. Under this weighting scheme, the PLCC values between the first-level comprehensive scores and the mAP50 values of YOLOv12n and RT-DETR-R18 are 0.685 and 0.656, respectively. The SRCC values are 0.850 and 0.826, respectively, and the Kendall’s tau values are 0.764 and 0.691, respectively. These results indicate that the first-level comprehensive scores show positive associations with the detection accuracy of both YOLOv12n and RT-DETR-R18. Therefore, the fourth weight combination is selected as the recommended first-level weight combination in this study, namely: $w_{env}=0.250$, $w_{quality}=0.125$, $w_{scale}=0.250$, and $w_{ann}=0.375$.

The weight allocation results show that dataset image annotation quality has the highest weight, namely 0.375. This indicates that factors such as annotation accuracy, annotation completeness, annotation consistency, and category annotation correctness have the most direct influence on the performance of object detection models under the current experimental conditions. The weights of dataset scale and acquisition environment are both 0.250, indicating that sample sufficiency, class distribution, illumination stability, background complexity, and object spatial distribution also affect model detection performance. The weight of image quality is 0.125. Its relatively low weight does not mean that image quality is unimportant for model training. Instead, it indicates that, among the eight datasets selected in this study, image quality indices have a relatively weaker explanatory ability for differences in detection accuracy than annotation quality, dataset scale, and acquisition environment. Accordingly, the first-level index scores of the datasets are calculated, as shown in Table 8.

**Figure 6:**
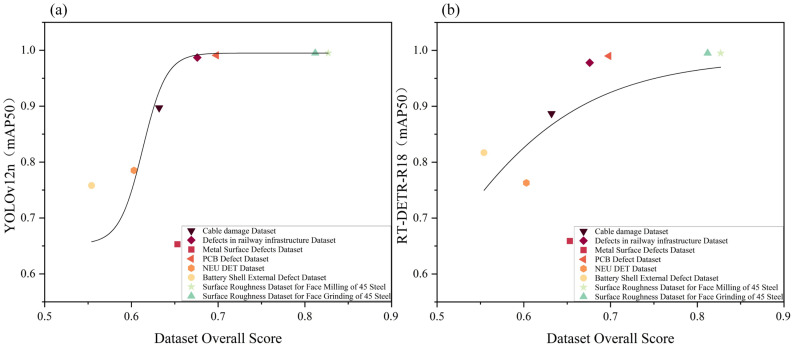
(**a**) Dataset overall score versus YOLOv12n mAP50. (**b**) Dataset overall score versus RT-DETR-R18 mAP50.

In Figure 6a,b, the scatter distributions and linear fitting results between the comprehensive scores of the first-level dataset indices and the detection accuracy of YOLOv12n and RT-DETR-R18 are presented, respectively. From the overall trend, as the comprehensive dataset score increases, the mAP50 values of both object detection models generally show an increasing trend. This suggests that the evaluation index system constructed in this study can reflect the supporting capability of datasets for object detection performance.

Although a positive correlation is observed between the proposed dataset quality score and the mAP50 values of the two detection models, the result should be interpreted carefully. The detection performance of a dataset is influenced not only by controllable quality factors, such as image quality, sample sufficiency, annotation reliability, and acquisition stability, but also by intrinsic task-difficulty factors, including object category characteristics, inter-class visual similarity, defect visibility, object scale, and scene complexity. Therefore, the reported PLCC, SRCC and Kendall’s tau values indicate an association between the comprehensive quality score and downstream detection performance.

### 4.3. Dataset Acquisition Guidelines

To further clarify the influence degree of the 13 s-level indices on the performance of industrial object detection models, this study conducts a main-effect analysis of the second-level indices based on the first-level weight analysis, using the 13 s-level indices as control factors in the Taguchi experiment. The PLCC and SRCC between the comprehensive scores of the second-level dataset indices and the mAP50 values of YOLOv12n and RT-DETR-R18 are used as evaluation criteria. To simultaneously consider linear correlation, ranking consistency, and cross-model stability, the comprehensive response value of the Taguchi experiment is defined, as shown in Equation (35).

(35) $$R=\frac{PLCC_{YOLOv12n}+SRCC_{YOLOv12n}+PLCC_{RT-DETR-R18}+SRCC_{RT-DETR-R18}}{4}$$

Here, *R* is used only as the response variable in the Taguchi experiment to compare the relative merits of different weight combinations. A larger *R* indicates stronger consistency between the comprehensive dataset evaluation score obtained under the corresponding weight combination and the performance of downstream detection models.

#### 4.3.1. Correlation Analysis Between Second-Level Indices and Detection Performance

Before the Taguchi main-effect analysis is conducted, the PLCC, SRCC, and corresponding *p*-values between each second-level index and the detection accuracy of YOLOv12n and RT-DETR-R18 are first calculated.

As shown in Table 9, the illumination stability, annotation completeness, annotation accuracy, MOS subjective image quality, and annotation consistency scores exhibit strong correlations with the mAP50 of both detection models. The PLCC and SRCC between the illumination stability score and YOLOv12n are reported as 0.856 and 0.898, respectively, with corresponding *p*-values both less than 0.01. This suggests that illumination stability may be an important factor affecting the performance of industrial object detection models. The annotation completeness score is associated with high SRCC values for both YOLOv12n and RT-DETR-R18, indicating that the completeness of target instance annotations directly affects the consistency between dataset quality ranking and detection performance ranking. Because multiple second-level indices are tested simultaneously in Table 9, these *p*-values are used only as nominal references for identifying potentially influential indices. They should not be interpreted as strict multiple-comparison-adjusted significance results. Therefore, the importance of each index is discussed together with its correlation magnitude, cross-model consistency, and its task relevance in industrial object detection.

It should be noted that a negative correlation is observed between the data integrity and standardization score and detection accuracy in this study. This result does not indicate that data standardization is unimportant or that it negatively affects model performance. Instead, it may be attributed to the heterogeneity of the selected datasets. Some public datasets have relatively standardized file structures and annotation formats, but their acquisition environments, image quality, annotation reliability, or intrinsic detection difficulty may not necessarily be superior, which can lead to relatively low detection performance. Therefore, data integrity and standardization should be regarded as a basic constraint for dataset usability, reproducibility, and training stability, rather than as a primary index used alone to distinguish differences in detection performance.

#### 4.3.2. Taguchi Experimental Results Based on the Orthogonal Table

The specific influence of the 13 s-level indices on object detection performance is further clarified by using these indices as control factors based on the first-level index weight analysis, and three weight levels—low, medium, and high—are established. The control factors and level settings are shown in Table 10. Since this study includes 13 control factors and each factor has three levels, an orthogonal table $L_{27}(3^{13})$ is adopted for experimental design.

According to the $L_{27}(3^{13})$ orthogonal table, 27 groups of second-level index weight combinations are designed in this study. For each experiment, the comprehensive evaluation scores of the eight datasets are calculated, and the PLCC and SRCC between these scores and the detection accuracy of YOLOv12n and RT-DETR-R18 are further computed. Finally, the average of the four correlation coefficients is used as the comprehensive response value *R*, and the results are shown in Table 11.

It should be noted that the Taguchi-based weight optimization in this study is a constrained weight-screening process rather than an independent external validation procedure. The weights are selected from a limited number of predefined levels and then normalized, which restricts the degree of freedom of the optimization. However, because the weight selection and correlation validation are both based on the same eight datasets, the optimized correlation coefficients may contain a certain degree of optimistic bias. Therefore, the recommended weights obtained in this study should be interpreted as recommended settings under the current dataset conditions.

As shown in Table 11, the highest comprehensive response value is achieved in the 18th experiment, with *R* = 0.820. Under this weight combination, the PLCC and SRCC between the comprehensive dataset evaluation score and YOLOv12n are 0.736 and 0.922, respectively. For RT-DETR-R18, the PLCC and SRCC are 0.724 and 0.898, respectively. This indicates that this combination better balances linear correlation, ranking consistency, and cross-model stability.

#### 4.3.3. Main Effects and Significance Analysis of Second-Level Indices

As shown in Table 12, the annotation completeness score has the largest range, with (Δ = 0.054), and its *p*-value is 0.031, which is less than 0.05. This indicates that annotation completeness is the most significant key factor among the 13 s-level indices. It also suggests that whether target instances are completely annotated directly affects the model’s learning of target distribution and category features. The ranges of the PIQE objective image quality score, background complexity score, illumination stability score, and annotation accuracy score are also relatively large, indicating that they have certain effects on the comprehensive response value. Among them, the optimal weight levels of illumination stability and annotation accuracy are both Level 3, indicating that increasing their weights helps enhance the consistency between the comprehensive dataset score and model detection performance. By contrast, the optimal weight levels of PIQE and background complexity are both Level 1, indicating that these two indices are more suitable as auxiliary constraint indices. For the dataset scale dimension, the optimal level of the class sample sufficiency score is Level 3, and its influence is stronger than those of the effective target pixel area score and total sample size score. This suggests that, under the experimental conditions of this study, maintaining a reasonable and balanced class sample distribution is more important for improving the effectiveness of dataset quality evaluation than simply increasing the number of images or target pixel area. It should be noted that the 18th experiment represents the best-performing combination among the tested orthogonal trials, whereas the recommended combination in Table 13 is derived from the main-effect analysis by selecting the optimal level of each individual factor. Therefore, the two combinations are not necessarily identical.

According to the main-effect analysis results, the recommended level combination of the second-level indices: $A_{3}B_{1}C_{1}D_{1}E_{1}F_{2}G_{3}H_{1}I_{3}J_{3}K_{3}L_{3}M_{1}$ is obtained. After normalizing this combination, the recommended weights of the 13 s-level indices are obtained.

**Figure 7:**
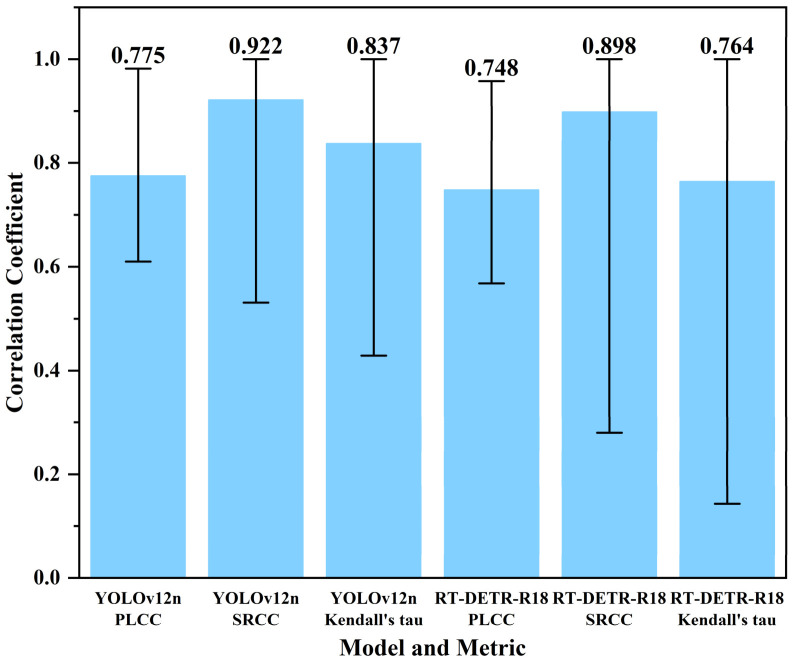
The 95% confidence intervals of PLCC, SRCC, and Kendall’s tau under the second-level weighting scheme.

Based on the recommended weights in Table 13, the comprehensive evaluation scores of the eight datasets are recalculated, and their correlations with the mAP50 values of the two detection models are verified. The uncertainty visualization of the correlation results is shown in Figure 7. Under the recommended second-level weighting scheme, the PLCC, SRCC, and Kendall’s tau between the comprehensive scores and the mAP50 values of YOLOv12n are 0.775, 0.922, and 0.837, respectively, with corresponding *p*-values of 0.024, 0.001, and 0.004. For RT-DETR-R18, the PLCC, SRCC, and Kendall’s tau are 0.748, 0.898, and 0.764, respectively, with corresponding *p*-values of 0.033, 0.002, and 0.009. These results indicate that the recommended weights of the second-level indices improve the consistency between the proposed comprehensive score and the detection performance of different object detection models.

The proposed comprehensive score can be calculated for a new dataset without using detection results, because all indices are derived from dataset properties such as acquisition environment, image quality, dataset scale, and annotation quality. Therefore, the score is intended to serve as a prior dataset quality assessment. However, the correlation analysis in this study is used to validate the association between the score and downstream performance on the selected datasets, rather than to establish an exact mAP prediction model.

To further visualize the uncertainty of the correlation results, the PLCC, SRCC, and Kendall’s tau values with 95% confidence intervals under the recommended second-level weighting scheme are presented in Figure 7. Compared with the first-level weighting scheme, the second-level weighting scheme results in higher PLCC, SRCC, and Kendall’s tau values for both YOLOv12n and RT-DETR-R18, indicating improved consistency between the comprehensive dataset quality score and downstream detection performance.

It should be noted that the correlation analysis is conducted at the dataset level. Therefore, although the selected datasets contain a large number of images, the effective number of validation samples for PLCC, SRCC, and Kendall’s tau is eight. To reduce the risk of over-interpreting a single correlation coefficient, PLCC is used to measure linear correlation, while SRCC and Kendall’s tau are used to evaluate rank-based consistency. In addition, two downstream detection models, YOLOv12n and RT-DETR-R18, are employed for cross-model validation, and 95% bootstrap confidence intervals are calculated to quantify the uncertainty of the correlation estimates. The reported *p*-values should be interpreted as nominal *p*-values and supplementary statistical references under the current small-sample setting.

Under the recommended second-level weights, the comprehensive dataset quality score shows improved consistency with the mAP50 values of both detection models. Specifically, both PLCC and SRCC increased compared with the first-level weighting scheme, and the Kendall’s tau values also indicated a consistent positive rank association. The bootstrap confidence intervals further provide an uncertainty range for these correlation estimates. Although the confidence intervals are relatively wide due to the limited number of datasets, the overall positive association trend remains consistent across YOLOv12n and RT-DETR-R18. These results suggest that the recommended second-level weighting scheme improves the agreement between the proposed dataset quality score and downstream detection performance under the current experimental conditions.

Since the same group of eight datasets is used for both weight selection and correlation validation, the optimized correlation coefficients may contain a certain degree of optimistic bias. In addition, only two detectors are included in this study, and the stability of the recommended weights under other detector architectures, random seeds, or independent dataset groups still requires further verification. Therefore, the reported *p*-values should be regarded as post-selection nominal *p*-values and supplementary statistical references. Accordingly, the main conclusion is based on the overall positive association trend, the correlation magnitudes, the confidence intervals, and the consistency of results across the two detection models.

#### 4.3.4. Sensitivity Analysis of Weighting Coefficients

To further evaluate the stability of the proposed scoring framework with respect to weight changes, a sensitivity analysis of the weighting coefficients is conducted. The recommended weights obtained by the Taguchi method are used as the baseline. Each weight coefficient is individually perturbed by ±10% and ±20%, while all weights are renormalized after each perturbation. The comprehensive dataset quality scores are then recalculated, and their PLCC, SRCC, and Kendall’s tau values with the mAP50 values of YOLOv12n and RT-DETR-R18 are recomputed.

**Figure 8:**
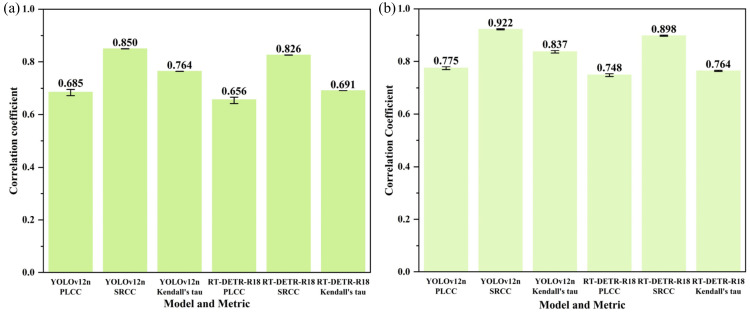
Sensitivity analysis of correlation coefficients under ±20% weight perturbation: (**a**) first-level weights; (**b**) second-level weights.

As shown in Figure 8, the correlation coefficients remain generally stable under moderate weight perturbations. Figure 8a presents the sensitivity analysis results for the first-level weights, while Figure 8b presents the results for the second-level weights. For the first-level weights, after ±20% perturbation, the PLCC of YOLOv12n varies from 0.672 to 0.696, while the SRCC and Kendall’s tau remained approximately 0.850 and 0.764, respectively. The PLCC of RT-DETR-R18 varied from 0.642 to 0.666, while the SRCC and Kendall’s tau remained approximately 0.826 and 0.691, respectively. For the recommended second-level weights, after ±20% perturbation, the PLCC of YOLOv12n varied from 0.769 to 0.780, while the SRCC and Kendall’s tau remained approximately 0.922 and 0.837, respectively. The PLCC of RT-DETR-R18 varied from 0.742 to 0.753, while the SRCC and Kendall’s tau remained approximately 0.898 and 0.764, respectively.

These results indicate that the positive association between the comprehensive dataset quality score and downstream detection performance is not substantially changed by moderate local perturbations of the recommended weights. In particular, the second-level weighting scheme maintains relatively high and stable correlations for both detection models, suggesting better robustness than the first-level weighting scheme. It should be noted that this sensitivity analysis only reflects local stability around the recommended weighting coefficients under the current dataset and model settings.

#### 4.3.5. Comparison with Simpler Evaluation Alternatives

To further compare the proposed comprehensive score with simpler evaluation alternatives, an ablation analysis of the first-level evaluation dimensions is conducted. In this analysis, acquisition environment, image quality, dataset scale, and image annotation are denoted as A, B, C, and D, respectively. All non-empty combinations of the four dimensions are calculated, and the corresponding dataset quality scores are recomputed after normalizing the weights within each combination. Then, the PLCC, SRCC, and Kendall’s tau values between these scores and the mAP50 values of YOLOv12n and RT-DETR-R18 are calculated. Considering the limitation of manuscript length, several representative schemes, including A, B, A + B, A + B + C, and the proposed comprehensive score, are selected for comparison, as shown in Table 14.

The results show that the single-dimension schemes have limited ability to explain the detection performance differences among datasets. For example, when only acquisition environment is used, the PLCC/SRCC/Kendall’s tau values are 0.501/0.539/0.400 for YOLOv12n and 0.545/0.587/0.473 for RT-DETR-R18. When only image quality is used, the corresponding values are 0.350/0.563/0.473 and 0.334/0.539/0.400, respectively. After combining acquisition environment and image quality, the correlations do not improve substantially, indicating that image-level acquisition and quality factors alone are insufficient to fully characterize dataset quality for industrial object detection. When dataset scale is further added, the correlations increase to 0.521/0.659/0.473 for YOLOv12n and 0.488/0.587/0.400 for RT-DETR-R18, suggesting that sample scale information contributes to the evaluation of dataset quality.

Compared with these single-dimension or partial-dimension schemes, the proposed comprehensive score achieves better overall consistency when PLCC, SRCC, Kendall’s tau, and cross-model stability are jointly considered. Its PLCC/SRCC/Kendall’s tau values are 0.685/0.850/0.764 for YOLOv12n and 0.656/0.826/0.691 for RT-DETR-R18. These results indicate that industrial dataset quality is jointly affected by multiple factors, including acquisition environment, image quality, dataset scale, and image annotation. Therefore, the proposed multidimensional evaluation framework provides a more comprehensive and stable assessment than single-dimension or partial-dimension alternatives.

## 5. Conclusions

Existing studies primarily conduct qualitative evaluations from a single dataset dimension, leading to limitations such as bias, low intuitiveness, and insufficient systematic analysis. Systematic and quantifiable assessments of dataset quality remain inadequate. Thus, a multidimensional quality evaluation system for industrial object detection image datasets is constructed in this study. The specific results are as follows:

(1) The eight datasets contain 41 categories and 20,811 images in total and cover several representative industrial scenarios. These datasets provide a preliminary basis for evaluating the feasibility of the proposed method. However, because the correlation analysis is conducted at the dataset level, the effective sample size is still limited. Therefore, further validation using more industrial datasets is needed in future work.
(2) The proposed system establishes first-level indices across four dimensions: acquisition environment, image quality, dataset scale, and image annotation. It introduces thirteen computable second-level indices, thereby transforming the evaluation of industrial image dataset quality from qualitative analysis to a comprehensive quantitative assessment.
(3) The weights of the four first-level indices are optimized using Taguchi orthogonal experiments. Results show that image annotation quality has the most significant impact on object detection performance, with a weight of 0.375. Both dataset acquisition environment and dataset scale are assigned weights of 0.250, while image quality has a weight of 0.125. YOLOv12n and RT-DETR-R18 are used as downstream detection models. After optimizing the first-level indices, the PLCC/SRCC/ Kendall’s tau values between the comprehensive scores and mAP50 for YOLOv12n and RT-DETR-R18 are 0.685/0.850/0.764 and 0.656/0.826/0.691, respectively.
(4) After further optimization of the 13 s-level indices using Taguchi orthogonal experiments, the PLCC/SRCC/Kendall’s tau values for YOLOv12n under the recommended weights are increased to 0.775/0.922/0.837, while those for RT-DETR-R18 reach 0.748/0.898/0.764, with PLCC and SRCC nominal *p*-values being less than 0.05. These results indicate that the dataset evaluation system constructed in this study effectively reflects the influence of industrial image dataset quality on detection model performance. Among the indices, annotation completeness, illumination stability, annotation accuracy, and class sample sufficiency are recognized as the key factors affecting detection performance. This finding is consistent with the weight distribution of the first-level indices.

It should be noted that the main novelty of this study lies in the integration, normalization, weighting, and downstream validation of existing and task-adapted indices within a unified dataset quality evaluation framework. The proposed framework is designed as a computable and reusable dataset quality assessment method for industrial object detection datasets. Since the indices are derived from intrinsic dataset attributes rather than from detection results, the framework can be applied to other industrial datasets for quality evaluation and comparison before model training.

Nevertheless, the present validation still has several limitations. The correlation analysis is conducted at the dataset level with only eight datasets, and the reported *p*-values should therefore be interpreted as supplementary statistical references. Although PLCC, SRCC, Kendall’s tau, sensitivity analysis, and two detection models jointly support the positive association between the proposed score and downstream detection performance, the quantitative predictive ability of the proposed score on unseen datasets still requires further external validation. Future work will include more datasets from different industrial domains, more detection models, and repeated experiments with different random seeds to further examine the robustness, generalizability, and practical applicability of the proposed framework.

## Figures and Tables

**Table 1:** Basic information of the datasets.

No.	Name	Categories	Descriptions	Sources
1	Cable Damage Dataset	2	This dataset contains two types of defects, break and thunderbolt. The dataset includes 1318 images in total, and the size of each image is 1528 × 1146.	https://universe.roboflow.com/roboflow-100/cable-damage (accessed on 22 February 2026)
2	Defects in Railway Infrastructure Dataset	4	This dataset contains railway images captured under normal operating conditions, including four categories, defective fishplate, fastener, missing fastener, and non-defective fishplate. It includes 1876 images in total, and the size of each image is 4000 × 3000.	https://universe.roboflow.com/rf20vl/defect-detection-yjplx-fxobh-amdi (accessed on 23 February 2026)
3	Metal Surface Defects Dataset	10	This dataset contains images collected from industrial production sites, including metal surface defects such as blowholes, weld defects, and crescent-shaped cracks. It contains 2280 grayscale images in total, and the size of each image is 2048 × 1000.	https://aistudio.baidu.com/datasetdetail/90446 (accessed on 23 February 2026)
4	PCB Defect Dataset	6	The dataset collects printed circuit board images from different manufacturers and different types, containing various defect types, including spur, short, and missing hole. The dataset contains 8001 images in total, and the size of each image is 600 × 600.	https://www.kaggle.com/datasets/norbertelter/pcb-defect-dataset (accessed on 17 February 2026)
5	NEU-DET Dataset	6	This dataset is created by the Northeastern University team, including six types of defects, crazing, patches, and scratches, among others. It contains 1800 images in total, and the size of each image is 200 × 200.	https://gitcode.com/open-source-toolkit/fa031 (accessed on 22 February 2026)
6	Battery Shell External Defect Dataset	3	This self-constructed dataset contains external surface defects of battery shells. It includes three types of defects, scratch, shallow pit, and deep concave. The dataset contains 2080 images in total, and the size of each image is 640 × 640.	https://pan.baidu.com/s/1DK0pwP0SjMdcfuy-H-LsLg?pwd=tw4f (accessed on 25 March 2026)
7	Surface Roughness Dataset for Face Milling of 45 Steel	6	This self-constructed dataset contains surface roughness images of 45 steel after face milling [18]. It includes six roughness categories, Ra 12.5 μm, Ra 6.3 μm, and Ra 3.2 μm, among others. The dataset contains 2176 images in total, and the size of each image is 2560 × 1920.	https://pan.baidu.com/s/1Ipx8vu9xIAVckZ-6YNXdeg?pwd=yzkd (accessed on 25 March 2026)
8	Surface Roughness Dataset for Face Grinding of 45 Steel	4	This self-constructed dataset contains surface roughness images of 45 steel after face grinding. It includes four roughness categories: Ra 0.8 μm, Ra 0.4 μm, Ra 0.2 μm, and Ra 0.1 μm. The dataset contains 1280 images in total, and the size of each image is 2590 × 1942.	https://pan.baidu.com/s/1i9IQyz-f7YZxiFSrTq4DTQ?pwd=vfqc (accessed on 25 March 2026)
Total		41 categories	20,811 images in total	

**Table 2:** Overall framework of the evaluation system proposed in this study.

First-Level Index	Second-Level Index	Calculation Formula	Value Range
acquisition environment	Illumination Stability	$S_{illum}=1-\frac{\sigma_{L}}{\mu_{L}}$	[0–1]
	Background Complexity	$S_{bg}=1-(w_{1}\frac{H}{H_{\max}}+w_{2}\frac{G}{G_{\max}}+w_{3}\frac{F}{F_{\max}})$	[0–1]
	Object Spatial Distribution Diversity	$S_{view}=\psi_{1}\cdot \frac{\sqrt{\sigma_{x}^{2}+\sigma_{y}^{2}}}{\sqrt{2}}+\psi_{2}\cdot C+\psi_{3}\cdot \frac{H_{s}}{H_{\max}}$	[0–1]
Image Quality	PIQE Objective Quality	$S_{PIQE}=1-\frac{PIQE}{100}$	[0–1]
	MOS Subjective Quality	$S_{MOS}=\frac{MOS-1}{4}$	[0–1]
Dataset Scale	Effective Target Pixel Area	$S_{res}=\min(1,\frac{\bar{A}}{A_{ref}})$	[0–1]
	Class Sample Sufficiency	$S_{cls}=1-\frac{1}{K}\sum_{i=1}^{K}\left|\frac{n_{i}-\bar{n}}{\bar{n}}\right|$	[0–1]
	Total Sample Size	$S_{vol}=\min(1,\frac{\ln(N_{eff}+1)}{\ln(N_{ref}+1)})$	[0–1]
Image Annotation	Annotation Completeness	$S_{comp}=\frac{N_{m}}{N_{r}+\lambda N_{fp}}$	[0–1]
	Annotation Accuracy	$S_{ann}=\frac{1}{N_{m}}\sum IoU_{i}\cdot \frac{N_{m}}{N_{r}}$	[0–1]
	Category Annotation Correctness	$S_{cat}=\sum_{k=1}^{K}w_{k}\frac{N_{c}^{\left(k\right)}}{N_{m}^{\left(k\right)}}$	[0–1]
	Annotation Consistency	$S_{cons}=\gamma S_{loc}+(1-\gamma )\kappa$	[0–1]
	Data Integrity and Standardization	$S_{data}=\beta_{1}S_{file}+\beta_{2}S_{pair}+\beta_{3}S_{format}+\beta_{4}S_{name}$	[0–1]

**Table 3:** Environment configuration.

Category	Specification
Operating System	Windows 11
CPU	Intel(R) Core(TM) i9-14900HX
Memory	32 GB
GPU	NVIDIA GeForce RTX 4060 Laptop GPU
CUDA Version	12.4
Python Version	3.11
PyCharm Version	2024.1
Deep Learning Framework	PyTorch-2.6.0

**Table 4:** Model training hyperparameter settings.

Hyperparameter	YOLOv12n	RT-DETR-R18
Epoch	200	200
Batch Size	16	4
Optimizer	AdamW	AdamW
Momentum	0.937	0.9
lr0	0.01	0.0001
Worker	0	4
Seed	0	0

**Table 5:** Normalized scores of first-level evaluation dimensions for each dataset.

No.	Dataset Name	Dataset Acquisition Environment	Dataset Image Quality	Dataset Scale	Dataset Image Annotation
1	Cable Damage Dataset	0.588	0.488	0.690	0.670
2	Defects in Railway Infrastructure Dataset	0.645	0.672	0.469	0.836
3	Metal Surface Defects Dataset	0.649	0.689	0.578	0.693
4	PCB Defect Dataset	0.641	0.677	0.664	0.765
5	NEU-DET Dataset	0.538	0.655	0.666	0.587
6	Battery Shell External Defect Dataset	0.614	0.581	0.477	0.557
7	Surface Roughness Dataset for Face Milling of 45 Steel	**0.757**	**0.801**	**0.745**	0.938
8	Surface Roughness Dataset for Face Grinding of 45 Steel	0.710	0.771	0.737	**0.944**

Note: Bold values indicate the highest normalized score in each first-level evaluation dimension.

**Table 6:** Control factors and levels for the Taguchi experiment of first-level indices.

Factors	Evaluation Indices	Level 1	Level 2	Level 3
A	Acquisition Environment	1	2	3
B	Image Quality	1	2	3
C	Dataset Scale	1	2	3
D	Image Annotation Quality	1	2	3

**Table 7:** Correlation analysis results of PLCC, SRCC, and Kendall’s tau for YOLOv12n and RT-DETR-R18.

No.	Experimental Condition	YOLO-PLCC	YOLO-SRCC	YOLO- Kendall’s Tau	RT-DETR-PLCC	RT-DETR -SRCC	RT-DETR -Kendall’s Tau
1	1-1-1-1	0.633	0.826	0.691	0.603	0.778	0.618
2	1-2-2-2	0.637	0.826	0.691	0.600	0.778	0.618
3	1-3-3-3	0.638	0.826	0.691	0.599	0.778	0.618
**4**	**2-1-2-3**	**0.685**	**0.850**	**0.764**	**0.656**	**0.826**	**0.691**
5	2-2-3-1	0.580	0.659	0.473	0.541	0.587	0.400
6	2-3-1-2	0.602	0.826	0.691	0.583	0.778	0.618
7	3-1-3-2	0.644	0.755	0.691	0.611	0.731	0.618
8	3-2-1-3	0.651	0.826	0.691	0.636	0.802	0.618
9	3-3-2-1	0.563	0.731	0.618	0.541	0.683	0.546

Note: Bold values indicate the optimal experimental condition and the corresponding highest correlation coefficients.

**Table 8:** Comprehensive scores of first-level dataset evaluation indices and accuracy of deep learning models.

No.	Dataset Name	Dataset Overall Score of First-Level Dataset Indices	YOLOv12n (mAP50)	RT-DETR-R18 (mAP50)
1	Cable Damage Dataset	0.632	0.897	0.887
2	Defects in Railway Infrastructure Dataset	0.676	0.987	0.978
3	Metal Surface Defects Dataset	0.653	0.653	0.659
4	PCB Defect Dataset	0.698	0.991	0.990
5	NEU-DET Dataset	0.603	0.785	0.763
6	Battery Shell External Defect Dataset	0.554	0.758	0.817
7	Surface Roughness Dataset for Face Milling of 45 Steel	0.827	0.995	0.995
8	Surface Roughness Dataset for Face Grinding of 45 Steel	0.812	0.995	0.995

**Table 9:** Correlations and *p*-values between second-level indices and detection model mAP50.

Second-Level Indices	YOLO-PLCC	*p*-Values	YOLO-SRCC	*p*-Values	RT-DETR-PLCC	*p*-Values	RT-DETRSRCC	*p*-Values
**Illumination Stability**	**0.856**	**0.007**	**0.898**	**0.002**	**0.795**	**0.018**	**0.874**	**0.005**
Background Complexity	−0.018	0.967	0.323	0.435	0.096	0.821	0.443	0.272
Object Spatial Distribution Diversity	−0.356	0.387	−0.383	0.349	−0.401	0.325	−0.407	0.317
PIQE Objective Quality	0.183	0.664	0.515	0.192	0.158	0.709	0.467	0.243
**MOS Subjective Quality**	**0.730**	**0.040**	**0.850**	**0.007**	**0.743**	**0.035**	**0.922**	**0.001**
Effective Target Pixel Area	−0.147	0.727	0.110	0.795	−0.184	0.663	0.061	0.885
Class Sample Sufficiency	0.357	0.385	0.755	0.031	0.372	0.364	0.731	0.040
Total Sample Size	0.207	0.623	0.188	0.655	0.054	0.900	0.047	0.912
**Annotation Completeness**	**0.817**	**0.013**	**0.898**	**0.002**	**0.790**	**0.020**	**0.874**	**0.005**
**Annotation Accuracy**	**0.746**	**0.034**	**0.898**	**0.002**	**0.727**	**0.041**	**0.874**	**0.005**
Category Annotation Correctness	0.357	0.385	0.755	0.031	0.372	0.364	0.731	0.040
**Annotation Consistency**	**0.751**	**0.032**	**0.826**	**0.011**	**0.719**	**0.045**	**0.802**	**0.017**
Data Integrity and Standardization	−0.811	0.015	−0.732	0.039	−0.778	0.023	−0.732	0.039

Note: Bold values indicate second-level indices that exhibit relatively strong and stable correlations with the mAP50 values of both YOLOv12n and RT-DETR-R18.

**Table 10:** Control factors and levels for the Taguchi experiment of second-level indices.

Factors	Second-Level Indices	Level 1	Level 2	Level 3
A	Illumination Stability	1	2	3
B	Background Complexity	1	2	3
C	Object Spatial Distribution Diversity	1	2	3
D	PIQE Objective Quality	1	2	3
E	MOS Subjective Quality	1	2	3
F	Effective Target Pixel Area	1	2	3
G	Class Sample Sufficiency	1	2	3
H	Total Sample Size	1	2	3
I	Annotation Completeness	1	2	3
J	Annotation Accuracy	1	2	3
K	Category Annotation Correctness	1	2	3
L	Annotation Consistency	1	2	3
M	Data Integrity and Standardization	1	2	3

**Table 11:** Taguchi orthogonal experimental results of second-level indices.

No.	Level Combination A–M	YOLO-PLCC	YOLO-SRCC	RT-DETR-PLCC	RT-DETR-SRCC	R
1	1-1-1-1-1-1-1-1-1-1-1-1-1	0.683	0.850	0.654	0.826	0.754
2	2-1-2-3-1-2-3-1-2-3-1-2-3	0.695	0.850	0.669	0.826	0.760
3	3-1-3-2-1-3-2-1-3-2-1-3-2	0.740	0.850	0.706	0.826	0.781
4	1-2-2-2-1-1-1-2-2-2-3-3-3	0.650	0.826	0.620	0.802	0.725
5	2-2-3-1-1-2-3-2-3-1-3-1-2	0.711	0.922	0.681	0.898	0.803
6	3-2-1-3-1-3-2-2-1-3-3-2-1	0.678	0.850	0.647	0.826	0.751
7	1-3-3-3-1-1-1-3-3-3-2-2-2	0.639	0.826	0.607	0.802	0.719
8	2-3-1-2-1-2-3-3-1-2-2-3-1	0.657	0.850	0.631	0.826	0.741
9	3-3-2-1-1-3-2-3-2-1-2-1-3	0.636	0.755	0.602	0.731	0.681
10	1-1-1-1-2-2-2-2-2-2-2-2-2	0.711	0.850	0.676	0.826	0.766
11	2-1-2-3-2-3-1-2-3-1-2-3-1	0.698	0.826	0.655	0.802	0.746
12	3-1-3-2-2-1-3-2-1-3-2-1-3	0.697	0.850	0.658	0.826	0.758
13	1-2-2-2-2-2-2-3-3-3-1-1-1	0.690	0.826	0.654	0.802	0.743
14	2-2-3-1-2-3-1-3-1-2-1-2-3	0.620	0.826	0.573	0.802	0.705
15	3-2-1-3-2-1-3-3-2-1-1-3-2	0.687	0.850	0.651	0.826	0.754
16	1-3-3-3-2-2-2-1-1-1-3-3-3	0.560	0.683	0.554	0.659	0.614
17	2-3-1-2-2-3-1-1-2-3-3-1-2	0.648	0.850	0.639	0.826	0.741
**18**	**3-3-2-1-2-1-3-1-3-2-3-2-1**	**0.736**	**0.922**	**0.724**	**0.898**	**0.820**
19	1-1-1-1-3-3-3-3-3-3-3-3-3	0.721	0.922	0.683	0.898	0.806
20	2-1-2-3-3-1-2-3-1-2-3-1-2	0.647	0.826	0.596	0.779	0.712
21	3-1-3-2-3-2-1-3-2-1-3-2-1	0.698	0.826	0.640	0.802	0.742
22	1-2-2-2-3-3-3-1-1-1-2-2-2	0.615	0.731	0.605	0.683	0.658
23	2-2-3-1-3-1-2-1-2-3-2-3-1	0.736	0.850	0.715	0.826	0.782
24	3-2-1-3-3-2-1-1-3-2-2-1-3	0.681	0.850	0.660	0.826	0.754
25	1-3-3-3-3-3-3-2-2-2-1-1-1	0.624	0.755	0.607	0.731	0.679
26	2-3-1-2-3-1-2-2-3-1-1-2-3	0.666	0.850	0.649	0.826	0.748
27	3-3-2-1-3-2-1-2-1-3-1-3-2	0.678	0.826	0.654	0.802	0.740

Note: Bold values indicate the optimal level combination and its corresponding correlation results, including the highest comprehensive response value.

**Table 12:** Main-effect and significance analysis results of second-level indices.

Second-Level Index	Mean at Level 1	Mean at Level 2	Mean at Level 3	Range Δ	Optimal Level	F-Value	*p*-Value	Contribution Rate (%)
Annotation Completeness	0.715	0.737	0.769	0.054	3	4.053	0.031	15.95
PIQE Objective Quality	0.762	0.737	0.721	0.041	1	2.043	0.152	12.11
Background Complexity	0.758	0.742	0.720	0.038	1	1.673	0.209	11.14
Illumination Stability	0.718	0.749	0.753	0.035	3	1.698	0.204	10.35
Annotation Accuracy	0.722	0.743	0.756	0.034	3	1.293	0.293	9.88
Object Spatial Distribution Diversity	0.757	0.732	0.731	0.026	1	0.964	0.396	7.59
Total Sample Size	0.752	0.740	0.728	0.025	1	0.660	0.526	7.29
Data Integrity and Standardization	0.751	0.742	0.728	0.023	1	0.568	0.574	6.75
Class Sample Sufficiency	0.736	0.731	0.753	0.023	3	0.595	0.559	6.65
Effective Target Pixel Area	0.740	0.746	0.734	0.013	2	0.161	0.852	3.67
Category Annotation Correctness	0.740	0.734	0.746	0.012	3	0.151	0.861	3.56
MOS Subjective Quality	0.746	0.739	0.736	0.010	1	0.114	0.893	3.00
Annotation Consistency	0.736	0.741	0.743	0.007	3	0.053	0.948	2.07

**Table 13:** Recommended weights of second-level indices.

No.	Second-Level Index	Recommended Level	Recommended Weight
1	Illumination Stability	3	0.115
2	Background Complexity	1	0.038
3	Object Spatial Distribution Diversity	1	0.038
4	PIQE Objective Quality	1	0.038
5	MOS Subjective Quality	1	0.038
6	Effective Target Pixel Area	2	0.077
7	Class Sample Sufficiency	3	0.115
8	Total Sample Size	1	0.038
9	Annotation Completeness	3	0.115
10	Annotation Accuracy	3	0.115
11	Category Annotation Correctness	3	0.115
12	Annotation Consistency	3	0.115
13	Data Integrity and Standardization	1	0.038

**Table 14:** Comparison with selected ablation schemes of first-level evaluation dimensions.

Evaluation Scheme	YOLOv12n PLCC	YOLOv12n SRCC	YOLOv12n Kendall’s Tau	RT-DETR-R18 PLCC	RT-DETR-R18 SRCC	RT-DETR-R18 Kendall’s Tau
A	0.501	0.539	0.400	0.545	0.587	0.473
B	0.350	0.563	0.473	0.334	0.539	0.400
A + B	0.466	0.515	0.327	0.485	0.539	0.400
A + B + C	0.521	0.659	0.473	0.488	0.587	0.400
Proposed comprehensive score	0.685	0.850	0.764	0.656	0.826	0.691

## Data Availability

The original contributions presented in this study are included in the article. Further inquiries can be directed to the corresponding author.
